# Surgical approach to uterine myomatosis in patients with infertility: open, laparoscopic, and robotic surgery; results according to the quantity of fibroids

**DOI:** 10.5935/1518-0557.20210049

**Published:** 2022

**Authors:** Héctor Salvador Godoy Morales, Radamés Rivas López, Germán Gabriel Palacios López, Pablo Joaquín Cervantes Mondragón, Daniel Vieyra Cortés, Hilda Sánchez Hernández, Miguel Loyo Guiot, Francisco Miguel Rojas Camacho

**Affiliations:** 1 Head of the Fertility Clinic at Ángeles del Pedregal Hospital, Lead Professor of the Human Reproductive Medicine Course, Robotic Gynecological Surgeon, Mexico City, Mexico; 2 Associate Professor of the Human Reproductive Medicine Course, Gynecological Robotic Surgeon, Ángeles del Pedregal Hospital, Mexico City, Mexico; 3 Specialist in Human Reproductive Medicine and Master in Health Sciences, Mexico City, Mexico; 4 Specialist in Human Reproductive Medicine, Mexico City, Mexico; 5 Associate Professor of the Human Reproductive Medicine Course, Ángeles del Pedregal Hospital, Mexico City, Mexico; 6 Resident of Human Reproductive Medicine at Ángeles del Pedregal Hospital, Mexico City, Mexico

**Keywords:** laparoscopic surgery, robotic surgery, myomectomy, infertility, fibroids

## Abstract

**Objective:**

To compare approaches to myomectomy (laparotomic, laparoscopic, and robotic). To show the relationship between the number of fibroids and the reproduction diagnosis.

**Methods:**

Observational, analytical, retrospective, and cross-sectional study; where the surgical approach used, was evaluated in terms of surgical bleeding, time, number and weight of fibroids and reproductive results.

**Results:**

69 patients were treated through different approaches and divided into 3 groups. The differences found among groups were in favor of laparotomic myomectomy in terms of the number (*p*=0.000) and weight of fibroids (*p*=0.004). Robotic surgery was also longer (*p*=0.000). In the analysis of the influence of the number of fibroids to achieve pregnancy, the result was in favor of the minimally invasive routes, after surgery, both in the group of < 6 fibroids (*p*=0.017), and that of > 6 fibroids (*p*=0.001), without differences in the time from surgery to pregnancy (*p*=0.979).

**Conclusions:**

The surgical approach decision should consider the number and size of resected fibroids, surgical time, and reproductive diagnosis. The minimally invasive route should be offered whenever possible due to its better outcome on achieving pregnancy, without forgetting the benefits of laparotomy, while also accrediting the recently introduced robotic-assisted approach.

## INTRODUCTION

Uterine myomas, also known as fibroids or leiomyomas, are the most common benign tumors in women of reproductive age (40% incidence, which can reach up to 70% at the age of 50) (Bulun, 2013). Depending on their location, number and size, the symptoms they produce may vary in frequency and severity. Although the relationship of fibroids to infertility is debatable, there is scientific evidence that fibroids interfere with sperm migration, oocyte transport and embryo implantation, due to the endometrial inflammation or the vascular alterations they cause (Khaund & Lumsden, 2008).

Approximately, 5 to 10% of infertile women are affected by fibroids, and their presence is the only abnormal fact found in 4% of them, i.e. without any other added pathology (Cook et al., 2010). The relationship between fibroids and fertility continues to be controversial, especially regarding intramural fibroids. It is generally accepted that submucosal fibroids decrease fertility, and that subserosal fibroids have little or null influence on this aspect (Klatsky et al., 2008). However, according to much of the medical literature, intramural fibroids affecting the endometrial cavity are also associated with adverse reproductive outcomes (Sunkara et al., 2010).

The impact of intramural fibroids that do not distort the endometrial cavity raises more questions, especially when choosing the most appropriate therapeutic strategy. A recent meta-analysis suggests that the presence of intramural fibroids that do not distort the cavity is associated with an adverse outcome in women undergoing in vitro fertilization (IVF) treatment (Sunkara et al., 2010). It is important to consider the rate of live births in addition to the post-IVF pregnancy rate, as fibroids can be associated with an adverse obstetric outcome. Furthermore, the rate of live births in patients with fibroids is 21% lower than that in women without fibroids.

Currently, in women of reproductive age with gestational desire and having fibroids, myomectomy is probably the treatment of choice, when the patient is a candidate for surgical treatment. Surgical strategy differs depending on the type of patient, especially when it comes to their desire to get pregnancy, it is important to emphasize that in patients with fibroids and infertility, pregnancy options should be maximized, while minimizing the risks arising from unnecessary myomectomies. It is advisable to make a distinction between reproductive-focused surgery and gynecological surgery, so that the surgical route of choice in patients with gestational desire should be endoscopic, whenever possible. Thus, hysteroscopic and laparoscopic myomectomy have become the gold standard of minimally invasive surgery (MIS).

Therefore, it could be said that, by consensus, surgery is indicated for submucosal and intramural fibroids affecting the cavity, but not for subserosal fibroids (Pritts et al., 2009). Current recommendations acknowledge that intramural fibroids greater than or equal to 4cm that do not affect the endometrial cavity should be removed (Galliano et al., 2015). In these cases, the route of choice for the surgical approach is laparoscopic versus hysteroscopic.

The surgical route of choice depends on two fundamental factors: the fibroid itself, its location, its number and size, and the experience and skills of the surgical team. Submucosal fibroids are treated hysteroscopically, while intramural and subserosal fibroids can be treated by laparoscopic, robotic, abdominal, or vaginal route. Whenever possible, the route of choice should be laparoscopic, since there is a solid scientific foundation which shows that through laparoscopic myomectomy there is less blood loss during surgery, a lower rate of adhesions, less postoperative morbidity and a reduced hospitalization time than with laparotomy and minilaparotomy myomectomy (Barakat et al., 2011); and even beyond, it enables better pregnancy and live birth rates in the group of patients undergoing laparoscopic myomectomy (Palomba et al., 2007).

Although conventional laparoscopic surgery has proven effective, robot-assisted laparoscopic surgery is a relatively recent innovation in the field of gynecologic surgery, which also has much to offer. Evidence shows that the MIS approach is feasible and safe for gynecological surgery, as much as it is a challenging procedure with a high degree of difficulty, and should ideally be performed by expert laparoscopists (AAGL Advancing Minimally Invasive Gynecology Worldwide, 2013). However, robotic myomectomy has not shown superiority compared to classic laparoscopic myomectomy yet, in terms of conventional postoperative outcomes.

Due to the absence of randomized studies that compare patients with infertility, the best surgical management and fertility prognosis is yet to be determined (Lonnerfors, 2018; Kim et al., 2018; Ascher-Walsh & Capes, 2010). For this reason, we decided to carry out a study to compare the techniques and reproductive outcomes, specifically referring to our population and thus add evidence concerning which route would be the most appropriate.

## MATERIAL AND METHODS

Observational, analytical, retrospective, and cross-sectional study in which we obtained the records of patients treated at the Fertility Clinic of the Angeles del Pedregal Hospital in Mexico City from 2010 to 2018, using the records of the clinical archive. Patients who had undergone "myomectomy" surgery by any type of approach were included in the study, and were subsequently divided into 3 groups depending on the implemented route: laparotomy, conventional laparoscopy, and robot-assisted laparoscopy. Within the inclusion criteria, they were patients of any age, with a weight that did not exceed grade I obesity, and patients without anemia prior to surgery (preoperative hemoglobin: <7 g/dl), with specification of the surgery indication (that is, they have been operated specifically for reproductive reasons, with uterine myomatosis as the only cause of infertility), regardless of the size, number and type of fibroids to be resected.

There were only two surgeons who performed the surgeries (the number of cases operated by each doctor were matched), and we analyzed the surgical time variables, the pregnancy rate after treatment and time to pregnancy/conception. Patients with insufficient information or those who did not seek pregnancy were excluded, as well as those that had ended in hysterectomy, in whom only bleeding complications and need for transfusion were analyzed. Similarly, patients who had received medical treatment prior to surgery (adjuvants) were excluded; although in all cases during surgery, intramyometrial injection of vasopressin was used (the administered dilution indicated by the treating physician was generally 1/50).

The information sources for the study were the clinical history obtained from the patients' charts and an ad hoc structured and designed personal epidemiological questionnaire. The sampling was of a conventional non-probabilistic design, until an equal number of cases per group was achieved (since robotic surgery is relatively new and there are few patients treated by such route). The research method used was non-participant observation and data collection through the information collection card.

For the statistical analysis of the results we used data processing software (Microsoft Excel^©^ and IBM SPSS Statistics^©^, Version 20) for descriptive and inferential statistics, using measures of central tendency and dispersion for quantitative variables, and with frequencies and proportions for the qualitative variables. Likewise, for the correlation calculation between variables, we used the Pearson correlation for quantitative variables with normal distribution; Spearman's correlation for those that did not have it; the comparison tests of means for independent variables; one-way ANOVA for quantitative variables of normal distribution, or otherwise Kruskal Wallis (differences between 3 groups). To predict if the surgical time and the time to achieve spontaneous pregnancy were influenced by other factors associated with the patient and the treatment, we used a simple linear regression; and finally, to obtain the differences in qualitative variables between the different approaches, we used the Fisher's exact test or the Chi-Square. This study was authorized by the Research Committee of the Ángeles del Pedregal Hospital and is in strict compliance with the current guidelines of the General Health Law in Chapter I of the Ethical Aspects of Human Beings Research, Article 17. The authors declare they have no conflicts of interest.

## RESULTS

We analyzed 69 patients treated by different myomectomy approaches, obtaining 3 groups: group 1) 21 cases underwent laparotomy; group 2) 24 cases underwent laparoscopy; and group 3) 24 cases had robotic assistance. The mean age of the patients was 36.43 (±4.85), with an average BMI of 24.47 (±3.19), and average preoperative hemoglobin of 10.53 (±5.05), with significant differences between groups only in BMI (p=0.04). Table 1 shows the demographic characteristics of the patients, which also shows that 21% of the patients had previously undergone myomectomy.

**Table 1.** General characteristics of the population.

Variable	Laparotomy	Conventional Laparoscopy	Robot-assisted Laparoscopy	*p*
Age (years)	36.9±4.5 34.9-39	37.24±5.65 34.9-39.5	35.23±4.19 33.5-36.9	0.287
BMI (kg/m^2^)	25.64±4.03 23.81-27.49	24.62±3.28 23.26-25.97	23.38±1.77 22.67-24.1	0.049
Preoperative Hb (g/dl)	10.25±5.3 7.83-12.6	9.96±5.3 7.77-12.1	11.31±4.66 9.42-13.1	0.614
Myomectomy History	28.5%	12%	23.07%	0.363

During the first part of the study, we evaluated the main characteristics of the 3 different approach types and their complications, according to the fibroid classification of the International Federation of Gynecology and Obstetrics (FIGO). One patient had a "Submucosal" fibroid; 49 patients had the "other kind"; and 16 patients had the "hybrid" type; without finding statistically significant differences between the groups (p=0.69); the maximum fibroid size was 6.3 cm in average (±5.75), and the "total" average fibroid weight was 162.34 grams (±731.94) per surgical event, finding statistically significant differences between the groups in the number (p=0.000) and weight (p 0.004) of fibroids , in favor of laparotomic myomectomy. Table 2 shows the rest of the corresponding surgical findings.

**Table 2.** Surgical findings.

Variable	Laparotomy	Conventional Laparoscopy	Robot-assisted Laparoscopy	*p*
Number of resected fibroids	9.24±8.7 5.25-13.23	2.56±1.7 1.85-3.27	3.85±3.06 2.6-5.09	0.000
Largest size (cm)	9.69±7.91 6.01-13.22	4.22±2.99 2.98-5.45	5.61±4.63 3.74-7.49	0.004
Fibroid weight (gr)	482.86±1307 112.2-1077.98	21.6±67.5 6.37-49.57	33.84±76.70 2.18-65.5	0.055
Most common fibroid type (FIGO)	Intramural (33.3%)	Intramural (32%)	Intramural (69.23%)	0.069

Within the eventualities and/or complications between the 3 different approach types, we could observe that the mean bleeding amount was 299 ml (±516.17), without statistically significant differences (*p*=.097) between the groups; but as to surgical time which was much longer in robotic surgery (*p*=0.000), with an average of 189 minutes (±94.07). In the same way, there were no differences in need for conversion in the MIS routes (*p*=0.399), nor in the need for transfusion (*p*=0.17) or days of hospital stay (0.525), these findings are described in Table 3, below.

**Table 3.** Eventualities and complications in the 3 approach routes.

Variable	Laparotomy	Conventional Laparoscopy	Robot-assisted Laparoscopy	*p*
Intraoperative bleeding (ml)	502.86±733.05 169.17-836.54	224±392.14 62.13-385.87	206.54±360.17 61.06-352.02	0.097
Need for transfusion (patient percentage)	14.28%	8%	3.84%	0.170
Surgical time	42.86±115.24 112.2-1077.98	47.08±98.3 55.5-88.6	189.85±94.07 151.8-227.8	0.000
Length of hospital stay (days)	2.1±.301 1.96-2.23	1.88±.600 1.63-2.13	2±.849 1.66-2.14	0.525
Complications	Bladder injury (4%)	None	None	*
Conversion	NA	8%	4%	0.399

* Non applicable

When analyzing whether the number of fibroids is associated with a greater amount of bleeding, we obtained a Spearman correlation index of -0.007 (*p* 0.954); indicating that, at least in this study group, the number of fibroids did not influence bleeding severity, regardless of the surgical approach. Similarly, when analyzing whether the number of fibroids, the surgical approach or the fibroid weight predicted surgical time, we ran a linear regression analysis (R2 of 0.317, ANOVA 10.21, gl 3, *p*=0.000), concluding that in 30% of cases these parameters determine surgical time, which is statistically significant in this group of patients.

In the second part of the study and in terms of reproductive outcomes, the pregnancy rate for each technique was: 23.8% for open, 33.3% for laparoscopic and 41.6% for robotic-assisted myomectomy. The different types of pregnancy depending on the surgical technique are shown in Table 4, where it is interesting to notice that there was a higher number of live births after robotic myomectomy, even though no statistically significant difference was found, either by type of pregnancy or by surgical approach (*p*<0.05).

**Table 4.** Types of pregnancy after myomectomy, depending on the surgical technique.

Type of Pregnancy	Laparotomy	Conventional Laparoscopy	Robot-assisted Laparoscopy	Total	*p*
None	16	16	14	46	0.876
Biochemical	2	1	3	6	---
In progress	0	3	2	5	---
Live birth	2	3	4	9	0.744
Miscarriage	1	1	1	3	---
Total of cases	21	24	24	69	---
Pregnancy rates	23.8%	33.3%	41.6%	---	---

Concerning the influence of the number of fibroids to achieve spontaneous pregnancy, the pregnancy rate with 6 or more removed leiomyomas decreased (35% in less than 6 fibroids versus 29% in more than 6 fibroids), which may even influence the absence of a prior pregnancy, and the achievement of post-surgery pregnancy. We could see this in the groups and as described in Table 5, there are statistically significant differences according to the different approach routes and the number of fibroids, in favor of the MIS, both conventional laparoscopic and robotic, after surgery in the groups with < 6 fibroids (*p*=0.017), and with > 6 fibroids (*p*=0.001), although without any differences in the time from surgery to pregnancy (*p*=0.979).

**Table 5.** Percentage of pregnancy before and after myomectomy, according to the number of fibroids (< / > 6) and depending on the surgical approach.

Variable	Laparotomy	Conventional Laparoscopy	Robot-assistedLaparoscopy	*p*
Previous pregnancy and <6 fibroids	9.5%	37.5%	25%	0.098
Previous pregnancy and >6 fibroids	4.76%	4.16%	12.5%	0.001
No previous pregnancy and <6 fibroids	42.85%	62.5%	58.3%	0.009
No previous pregnancy and >6 fibroids	42.85%	0	12.5%	0.006
Post-surgery pregnancy and <6 fibroids	14.3%	29.16%	20.83%	0.017
Post-surgery pregnancy and >6 fibroids	9.52%	4.16%	12.5%	0.001
Time from surgery to pregnancy (years)	3.61±7.40 .248-6.99	3.91±6.63 1.11-6.71	4.04±6.84 1.150-6.93	0.979

In general, the average time to achieve spontaneous pregnancy of 3.87 years (±6.84) was observed when performing the overall analysis. We concluded that intervention is better than conventional and expectant management, since the patients were diagnosed with infertility and some of the achieved pregnancy after the surgical procedure, although it would be ideal not to seek pregnancy in the short term, to improve the results, since our patients achieved spontaneous pregnancy after 3 years.

## DISCUSSION

Myomectomy is one of the most performed worldwide reproductive surgeries considering that up to 50% of patients will achieve postoperative pregnancy, and it is the preferred MIS route, supported by several studies in terms of the lesser postoperative pain, bleeding, intrahospital stay, smaller scars and prompt reintegration into daily activities (Hurst et al., 2005).

The first keypoint presented by this study presents is the appropriate approach for each patient according to the number, size and location of the fibroids. This is reaffirmed in a recent review that assesses the different types of approaches to fibroids, where it is mentioned that these parameters must always be considered before deciding on a surgical approach (Mas et al., 2017). The previous assessment of such parameters will help determine which approach is superior, finding that generally the number and size of resected fibroids is higher for open surgery than for laparoscopic and robotic surgery. Open surgery was preferred in a time when it was considered safer to perform abdominal myomectomies, whereas, according to our recent study results, through the MIS approach there is a higher pregnancy rate, as shown in Table 5.

Other parameters that gained relevance when analyzed were the eventualities and complications, as per approach route. Regarding the amount of surgical bleeding, there were no differences between the 3 types of myomectomy (although at first glance it was higher in laparotomy). In some cases, transfusion was needed, which was apparently related to the larger number and size of fibroids. These data differ from others where intraoperative hemorrhage has been alluded to be less with minimal invasion than with open surgery (110 vs. 340 ml) (Ascher-Walsh & Capes, 2010; Palomba et al., 2007).

As for the surgical time depending on the approach, there were general differences, but robotic surgery had the worst record, which is consistent with most of the studies reported in the literature. The minimally invasive routes, despite not requiring so much time to close wounds, have the disadvantage of requiring access for fibroid extraction, or in the case of robotic surgery, a time for device engagement, which is time-consuming (Barakat et al., 2011), and in the long run also represents a higher cost. Even if the latter parameter was not evaluated in our study, it has already been widely discussed in others (Advincula et al., 2007; Behera et al., 2012).

As the second keypoint of this study, we identified that the number of fibroids influenced pregnancy achievement before and after surgery, regardless of the type of approach, even though this study is in favor of an MIS approach, especially with robotic assistance. Our findings are consistent with other studies that reported that, the higher the number of fibroids, the lower the rate of pregnancy. Nevertheless, in some other studies, the open surgery technique for multiple fibroids is preferred, as it ensures a shorter surgical time, and it is widely known and used especially by less experienced surgeons in MIS (Stringer et al., 1997; Tan et al., 2008). The surgeries carried out in this study were performed by surgeons with extensive experience and training in all approach types, and this may have influenced the outcomes, since even in the complicated cases addressed by MIS procedures, as many fibroids as possible were resected, despite the complexity of the technique.

Finally, in terms of post-surgery reproductive outcomes, the pregnancy rate was higher for robotic-assisted surgery and even for conventional laparoscopic surgery; hence finding statistically significant data. Surgical intervention is recommended as well as avoidance of short-term pregnancy. These results are consistent with other studies, which highlighted the importance of performing surgery instead of conventional management (non-intervention), considering the necessity to tailor the patient's case according to fibroid size and type, as well as infertility (Donnez & Jadoul, 2002; Griffiths et al., 2006; Khalaf et al., 2006).

Additionally, it would be interesting to analyze the postoperative complications of our cases, such as postoperative adhesions, uterine rupture (which at the time of the closing the patient search, was not found in any of the treated patients, or at least there was no knowledge or record of any case), infections, etc., since it has been noticed that in the MIS approach, postoperative adhesions are less frequent than uterine rupture or even infections (Seracchioli et al., 2000; Dubuisson et al., 2000; Palerme & Friedman, 1966; Garnet, 1964). We did not find any relevant differences between MIS approaches (conventional laparoscopic surgery and robotic-assisted surgery). However, the robotic-assisted surgery increases the surgical productivity of the specialist and it is more precise and efficient to perform uterine sutures with more comfort and ease for some cases that are of greater technical difficulty (Rivas-López et al., 2016).

## CONCLUSIONS

The surgical approach is closely related to the number and size of resected fibroids, surgical time, and reproductive diagnosis. If we take into consideration the patient's number of fibroids as well as the advantages and disadvantages of each approach, optimal outcomes can be obtained. An experienced surgeon with competent training can offer, with greater certainty, the preferred approach and, whenever possible, the MIS route with better chances in reproductive success (to achieve pregnancy).

Moreover, the fact that the bleeding severity with these approaches is not increased and they grant the possibility of offering the patient safe areas when performing the myomectomy, which, in terms of aesthetics, recovery time and especially reincorporation to useful life, has indeed proven to be clear advantages (in laparoscopic and robotic surgery). Even after the above comparison, the benefits of a laparotomy should not be forgotten, such as shorter surgical time and the possibility of managing complicated cases by a less experienced or trained surgeon.

Within the limitations of the study, there is its retrospective nature and the few number of cases; nevertheless, it is one of the greatest series in Mexico, in which it was possible to compare the 3 approaches, giving the patient - according to our medical criterion - to choose the surgery of preference based on the data found in the present study and other medical literature.
